# *MTHFR* Gene Polymorphisms and Cancer Risk in Children and Adolescents: A Systematic Review and Meta-Analysis

**DOI:** 10.3390/children12010108

**Published:** 2025-01-17

**Authors:** Savvas Kolanis, Eleni P. Kotanidou, Vasiliki Rengina Tsinopoulou, Elisavet Georgiou, Emmanuel Hatzipantelis, Liana Fidani, Assimina Galli-Tsinopoulou

**Affiliations:** 1 2nd Department of Paediatrics, School of Medicine, Faculty of Health Sciences, Aristotle University of Thessaloniki, AHEPA University Hospital, 54636 Thessaloniki, Greece; savvasks@auth.gr (S.K.); epkotanidou@auth.gr (E.P.K.); vitsinop@auth.gr (V.R.T.); hatzip@auth.gr (E.H.); sfidani@auth.gr (L.F.); 2 Laboratory of Biological Chemistry, School of Medicine, Faculty of Health Sciences, Aristotle University of Thessaloniki, 54124 Thessaloniki, Greece; elgeorgiou@auth.gr; 3 Laboratory of Medical Biology-Genetics, School of Medicine, Faculty of Health Sciences, Aristotle University of Thessaloniki, 54124 Thessaloniki, Greece

**Keywords:** *MTHFR* gene, methylenetetrahydrofolate reductase, single-nucleotide polymorphism, cancer, neoplasms, leukemia, child, adolescent

## Abstract

**Background/Objectives:** *MTHFR* gene polymorphisms (677C>T and 1298A>C) correlate with various types of cancer across all age groups; however, a small number of studies have included solely children and adolescents. The aim of this systematic review and meta-analysis was to present and synthesize all the available evidence on the association between *MTHFR* gene polymorphisms and the incidence of all types of cancer in children and adolescences. **Methods:** After a systematic search of all of the available data, original case–control studies involving children or adolescents with a confirmed diagnosis of any type of cancer and a molecular genetic test of *MTHFR* gene polymorphisms were included. **Results:** A total of 53 original studies in children and adolescents with cancer were included in the systematic review. Among these, 40 studies reviewed children and adolescents with Acute Lymphoblastic Leukemia (ALL), 4 those with Acute Myeloblastic Leukemia (AML), 8 those with central nervous system (CNS) tumors and 3 those with other types of cancer. Children and adolescents with ALL had less frequent T allele sequences (CT and TT variations) of the 677C>T polymorphism compared to a healthy population (OR: 0.85; CI: 0.80–0.91; *p* < 0.00001). Concerning the 1298A>C polymorphism, the C allele sequences (AC and CC) did not present a statistically significant difference in frequency compared to a healthy population (OR: 1.01; CI: 0.95–1.08; *p* = 0.69). **Conclusions:** Children and adolescents with ALL appeared to have the T allele sequences of the 677C>T polymorphism of the *MTHFR* gene less frequently compared to a healthy population.

## 1. Introduction

The 5,10-methylenetetrahydrofolate reductase (*MTHFR*) gene, located on the short arm of chromosome 1, produces a 656-amino-acid protein of paramount importance in folate metabolism and therefore in deoxyribonucleic acid (DNA) methylation [1,2,3]. This enzyme catalyzes the reductive reaction of 5,10-MethylTHF into 5-MethylTHF, which is the only source of 5-MethylTHF (the biologically active form of folate) in cells and is additionally used as a co-substrate for the remethylation of homocysteine into methionine. The 5-10 *MTHFR* enzyme therefore plays a central role in the metabolism of homocysteine (Hcy) and of folic acid, which is even more evident, as mutations and polymorphisms of the *MTHFR* gene lead to hyperhomocysteinemia and reduced levels of 5-MethylTHF [4,5].

Hundreds of *MTHFR* gene polymorphisms have been discovered in recent decades. The ClinVar archive by the National Institute of Health reports 945 variations in the *MTHFR* gene, 93 of which are registered as pathogenic [6]. The three most common polymorphisms are located at nucleotide positions 677, 1298 and 1317. The single-nucleotide polymorphism (SNP) 677C>T results in a heat-sensitive variant of the final protein product with reduced enzyme activity. This SNP is located in exon 4 of the *MTHFR* gene, where cytosine is replaced by thymine, causing a change in the nature of the enzyme, as it results in the substitution of the amino acid valine for alanine. The SNP 1298A>C occurs in exon 7 within the region of the considered regulatory domain, as adenine is replaced by cytosine, also resulting in a change in the protein produced, with the conversion of glutamic acid into alanine. Finally, the third SNP 1317T>C is located in the same region, in exon 7, causing a silent mutation without a change in the function of the final product and without clinical significance [3,7,8].

The 677C>T polymorphism (rs1801133) of the *MTHFR* gene is one of the most common polymorphisms of the *MTHFR* gene, and many researchers have suggested that it leads to hyperhomocysteinemia. The 1000 Genomes Project reported that this particular polymorphism is recognized worldwide in approximately 25% of people, with the highest frequency in Hispanic and European populations (47% and 36%, respectively), followed by Asian populations (East Asia at 30% and South Asia at 12%) and with the lowest frequency in African populations (9%) [9,10,11]. This particular polymorphism is associated with compromised reductase activity, with this enzyme’s function and activity reported to be reduced by up to 50%, resulting in an increase in Hcy levels of approximately 30% [3,12]. This observation is mainly reported in individuals homozygous for this particular SNP (TT variant), with the lowest recorded enzyme activity and consequently the highest Hcy levels and reduced plasma folate levels [3,4]. Moreover, researchers suggest that supplementation with the active form of folate (5-MethylTHF) provides a protective effect in patients carrying this particular SNP, as their folate levels are normalized and the risk of diseases associated with hyperhomocysteinemia is minimized [13,14,15,16,17,18].

The 1298A>C (rs1801131) polymorphism does not appear to affect Hcy or folate levels. However, double heterozygosity for the 677C>T and 1298A>C alleles in individuals could be responsible for reduced plasma folate and an increased Hcy concentration and is also strongly implicated in renal failure and several other diseases [12,19].

*MTHFR* gene polymorphisms are associated with an increased incidence of various cancer and malignancies across all ages but are also associated with non-communicable diseases. The 677C>T polymorphism is associated with colon, breast, pancreas and stomach cancer and also with heart disease, hypertension, diabetes mellitus type 1 and type 2, hypothyroidism and other conditions such as autism spectrum disorder, birth defects, congenital heart disease, neonatal defects and Down’s syndrome. The 1298A>C polymorphism is associated with myocardial infarction, stroke, diabetic nephropathy, autism spectrum disorders and recurrent pregnancy loss [7,20,21,22,23,24,25,26,27,28,29,30,31,32,33,34,35,36,37,38,39,40,41]. The evidence regarding the association between Acute Lymphoblastic Leukemia (ALL) and *MTHFR* polymorphisms across all ages is inconsistent [42,43,44]. In addition, hyperhomocysteinemia, a condition often resulting from *MTHFR* gene polymorphisms, is an independent risk factor for several types of cancer [45]. Many studies have investigated *MTHFR* polymorphisms in relation to the incidence of the most common types of cancer across one’s lifespan. Childhood and adolescent cancers have distinct etiologies, genetic backgrounds, epidemiological distributions and microenvironmental characteristics compared to those of adult cancers [46,47].

The current systematic review and meta-analysis aimed to present and synthesize the available evidence on the relation between *MTHFR* polymorphisms and cancer incidence in children and adolescences.

## 2. Materials and Methods

The eligibility criteria included original case–control studies consisting of children or adolescents (up to 21 years of age). A confirmed diagnosis of any type of cancer was required in the patient group, and a molecular genetic test of *MTHFR* gene polymorphisms (confirmed using genetic testing, a Sanger analysis, direct sequencing or any other molecular method) was also required for all participants. The study protocol was registered in the International Prospective Register of Systematic Reviews (Prospero, Registration number: CRD42024564716). There was no restriction on the publication status of the candidate studies during the study selection. Only studies published in the English language were included.

Studies and registries were identified from the MEDLINE, Scopus, ScienceDirect, EBSCO, Cochrane Library and ClinicalTrials.gov databases. The database search was also restricted to studies published between March 1998 and July 2024. The search strategy in MEDLINE (PubMed) was (*MTHFR* or methylenetetrahydrofolate reductase) and (cancer or neoplasms) and (children or adolescents). The search strategy for ScienceDirect was (*MTHFR* gene OR *MTHFR*) AND (cancer OR malignancy) AND (children or adolescents). The search strategy for EBSCO was (“*MTHFR*” OR “methylenetetrahydrofolate reductase” OR “*MTHFR* gene polymorphisms” OR “677” OR “1298”) AND (“cancer” OR “malignancies” OR “neoplasms” OR “ALL”) AND (“children” OR “adolescent”). The search strategy for Cochrane Library was *MTHFR* gene or Methylenetetrahydrofolate Reductase. The search strategy for ClinicalTrials.gov was the use of *MTHFR* OR *MTHFR* gene mutations as the search filters.

A review management tool was used to assess eligible studies, and a prototype data extraction form was also created to categorize all of the extracted data. The selection process and the data extraction process were performed by two independent investigators (with screening and inclusion based on the eligibility criteria). All disagreements were resolved by a third party. The team of researchers was gathered prior to the study selection and data extraction phases and conducted pilot testing in an effort to minimize errors. No automation tools were used. The data extracted from the selected studies consisted of the following: study ID, published title, authors, date of publication and country of origin, population’s origin, type of cancer/malignancy, staging, genetic variance method, study’s funding. Sample size, gender (male/female), age (mean value and range), *MTHFR* gene polymorphism 677C>T variants (CC, CT, TT) and *MTHFR* gene polymorphism 1298A>C variants (AA, AC, CC) were recorded separately for the patient and control groups. When data were missing from a study, we used simple statistical methods (mean values, conversion of percentages and addition/subtraction) to obtain them, or we attempted to contact the authors to obtain the relevant missing data. This study’s design, protocol and methods were based on the Preferred Reporting Items for Systematic reviews and Meta-Analyses (PRISMA) statement [48] (Table S1 in Supplementary Materials).

A qualitative assessment of the case–control studies included was performed based on the Newcastle–Ottawa scale (NOS) star rating system, as recommended by the Cochrane Handbook for Systematic Reviews and Meta-analyses [49,50]. The evaluation assessed the 3 key perspectives in each study design: the selection of the participants in each group, the comparability of the groups and the ascertainment of exposure.

The primary outcome of this study was to report the incidence of *MTHFR* polymorphisms amongst children and adolescents with any type of diagnosed cancer or malignancy. The outcomes were sorted and presented separately per category. A subgroup analysis was performed based on the country and continent of the researched population. The risk of publication bias was managed using a funnel plot analysis.

### Data Synthesis and Statistical Analysis

Homogenous data were synthesized using the Comprehensive Meta-Analysis software and Review Manager software, RevMan v.5.4 by Cochrane Collaboration. A meta-analysis was conducted among studies in children and adolescents with ALL, as we assessed that the eligible studies were sufficiently homogeneous in design and comparison. In the other cancer categories, no further statistical synthesis was performed due to heterogeneity and/or due to an insufficient number of studies.

Dichotomous data were determined using odds ratios (Ors) with 95% confidence intervals (Cis). Continuous outcomes were analyzed using weighted mean differences (with the 95% CI) or standardized mean differences (95% CI) when different measurement scales were used. The Chi-square test and the I^2^ test were used to define heterogeneity. Statistical heterogeneity was tested using the Chi-square test (significance level: 0.1) and the I^2^ statistic. For high levels of heterogeneity (I^2^ ≥ 50% or *p* < 0.1), the study design and characteristics were evaluated in the included studies as suggested by the Cochrane Handbook for Systematic Reviews. We performed a subgroup analysis based on the populations’ origins. In order to control for publication bias (meta-bias), a funnel plot was applied, and Egger’s regression test (publication bias if *p* < 0.05) was performed [51].

## 3. Results

### 3.1. Acute Lymphoblastic Leukemia (ALL)

**Figure 1:**
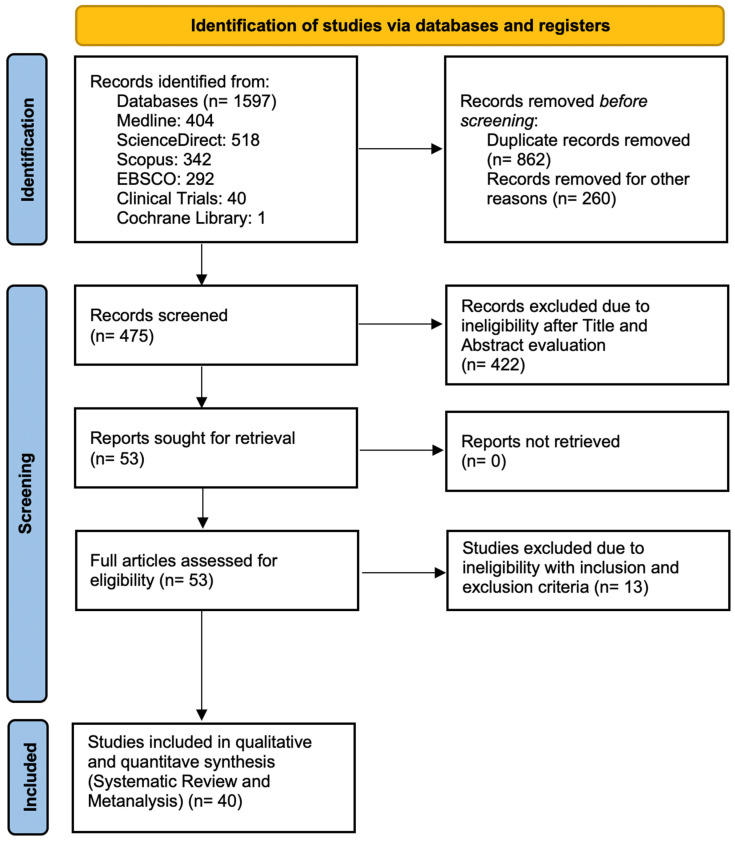
Flow chart diagram for studies that included children and adolescents with ALL.

A total of 1597 studies were reviewed through databases and registries. After removing duplicate studies, the remaining records were assessed for eligibility according to the inclusion and exclusion criteria, as shown in the flow chart (Figure 1). The search concluded with a total of 50 articles being reviewed at full-text level. A total of 13 articles were excluded for different reasons [52,53,54,55,56,57,58,59,60,61,62,63,64]. The final qualitative and quantitative synthesis included 40 original studies with a total of population of 7704 ALL patients and 10,825 control participants [65,66,67,68,69,70,71,72,73,74,75,76,77,78,79,80,81,82,83,84,85,86,87,88,89,90,91,92,93,94,95,96,97,98,99,100,101,102,103,104] (Table S2 in Supplementary Materials).

Male participants were more numerous than females in both the patient and control groups in the included studies (56.83% vs. 43.17% in patient groups and 53.11% vs. 46.89% in control groups).

**Figure 2:**
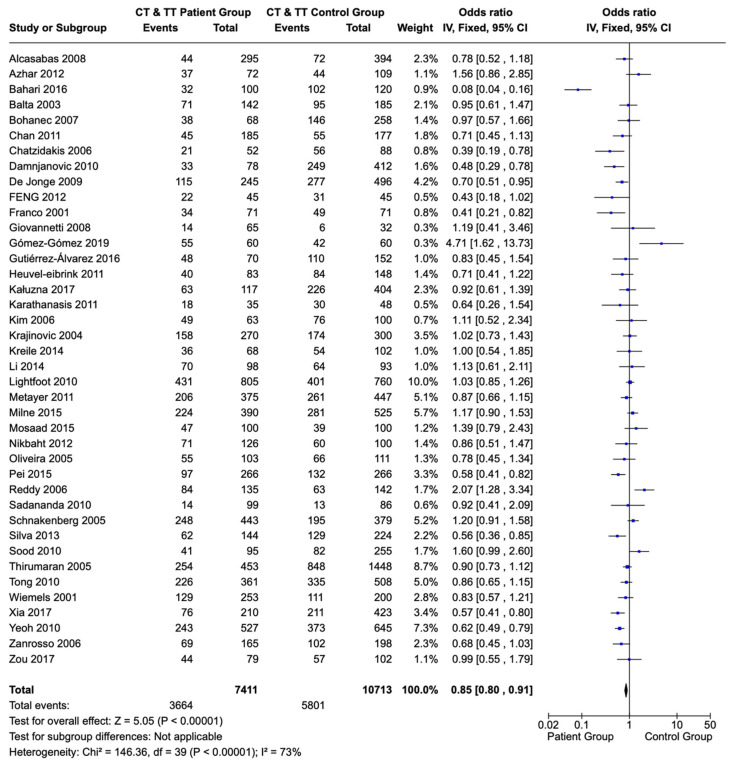
Forest plot of the correlation of T allele sequences (CT and TT variations) of the 677C>T polymorphism of the *MTHFR* gene in children and adolescents with ALL versus a healthy population. Each individual study is portrayed by a box (effect estimate) and a horizontal line (length of confidence interval) in the right column. The diamond shows the pooled result [65,66,67,68,69,70,71,72,73,74,75,76,77,78,79,80,81,82,83,84,85,86,87,88,89,90,91,92,93,94,95,96,97,98,99,100,101,102,103,104].

The statistical synthesis recorded the incidence of the *MTHFR* polymorphisms (677C>T and 1298A>C) amongst children and adolescents with the diagnosis of ALL. Children and adolescents with ALL presented with the T allele sequences (CT and TT variations) of the 677C>T polymorphism of the *MTHFR* gene less frequently when compared with a healthy population (OR: 0.85; CI: 0.80–0.91; *p* < 0.00001) (Figure 2).

**Figure 3:**
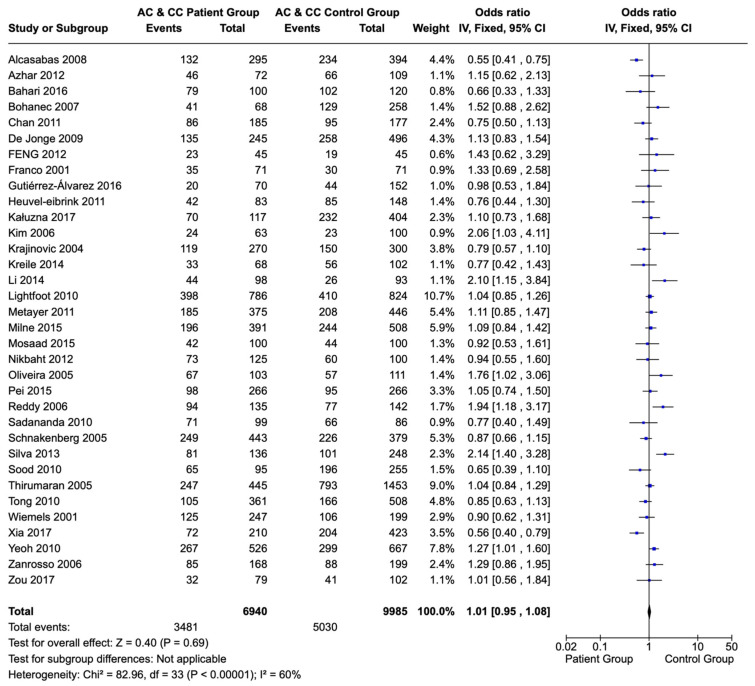
Forest plot of the correlation of C allele sequences (AC and CC variations) of the 1298A>C polymorphism of the *MTHFR* gene in children and adolescents with ALL versus a healthy population. Each individual study is portrayed by a box (effect estimate) and a horizontal line (length of confidence interval) in the right column. The diamond shows the pooled result [65,66,67,68,69,70,71,72,73,74,75,76,77,78,79,80,81,82,83,84,85,86,87,88,89,90,91,92,93,94,95,96,97,98,99,100,101,102,103,104].

Additionally, children and adolescents with ALL and the C allele sequences (AC and CC) of the 1298A>C polymorphism of the *MTHFR* gene did not present a statistically significant difference in their frequency compared to that in a healthy population (OR: 1.01; CI: 0.95–1.08; *p* = 0.69) (Figure 3).

**Figure 4:**
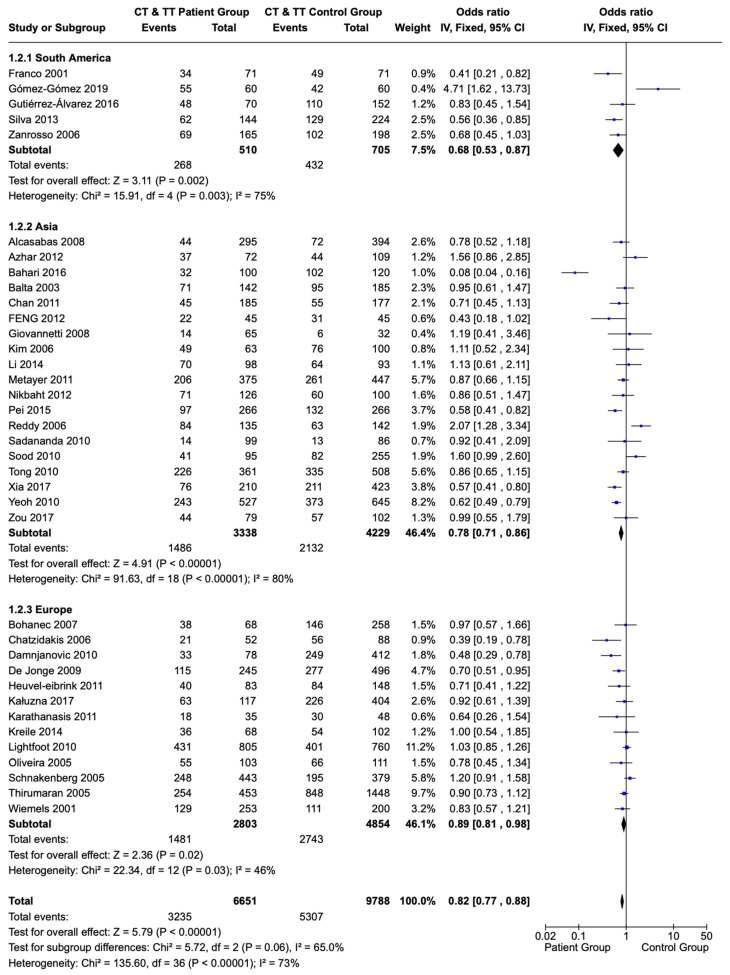
Forest plot and subgroup analysis of the correlation of T allele sequences (CT and TT variations) of the 677C>T polymorphism of the *MTHFR* gene in children and adolescents with ALL versus a healthy population based on the population’s origin. Each individual study is portrayed by a box (effect estimate) and a horizontal line (length of confidence interval) in the right column. The diamond shows the pooled result [65,66,67,68,69,70,71,72,73,74,75,76,77,78,79,80,81,82,84,85,86,87,90,91,92,93,94,95,96,97,98,99,100,101,102,103,104].

A subgroup analysis of the 677C>T polymorphism showed results consistent with the analysis of the entire group. Children and adolescents with ALL originating from South American countries had T allele sequences (CT and TT) of the 677C>T polymorphism of the *MTHFR* gene less frequently compared with a healthy population of the same origin (OR: 0.68; CI: 0.53–0.87; *p* = 0.002). Similar results were also observed in children and adolescents originating from Asia and Europe (OR: 0.78; CI: 0.71–0.86; and *p* < 0.00001 and OR: 0.89; CI: 0.81–0.98; and *p* = 0.02, respectively) (Figure 4).

**Figure 5:**
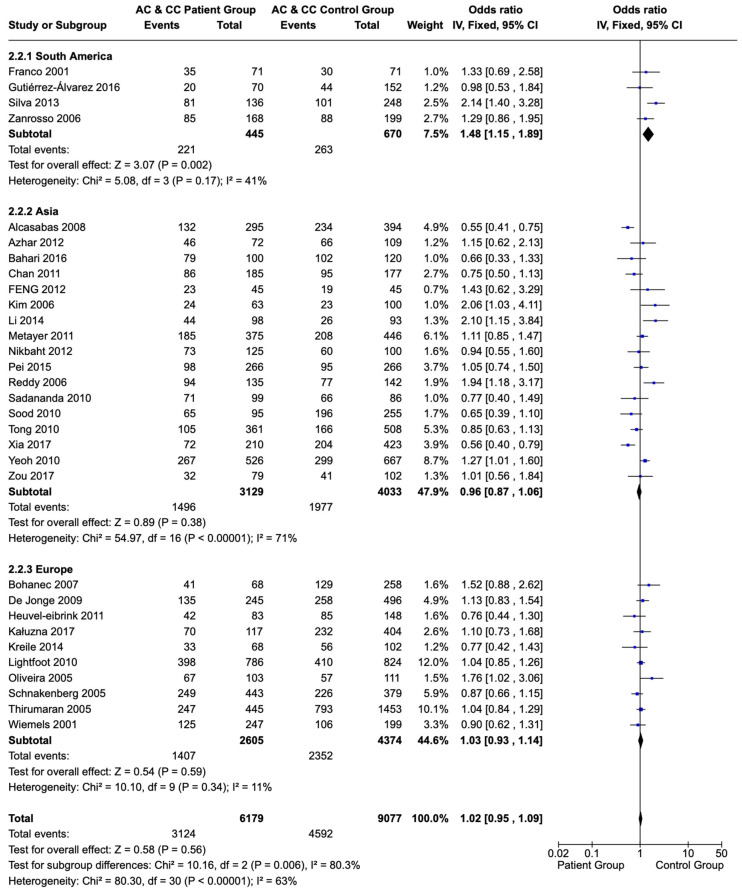
Forest plot and subgroup analysis of the correlation of C allele sequences (AC and CC variations) of the 1298A>C polymorphism of the *MTHFR* gene in children and adolescents with ALL versus a healthy population based on the population’s origin. Each individual study is portrayed by a box (effect estimate) and a horizontal line (length of confidence interval) in the right column. The diamond shows the pooled result [65,66,67,68,69,70,71,72,73,74,75,76,77,78,79,80,81,82,84,85,86,87,90,91,92,93,94,95,96,97,98,99,100,101,102,103,104].

A subgroup analysis of the 1298A>C polymorphism of the *MTHFR* gene did not show a significant difference in the frequency of C allele sequences (AC and CC) in children and adolescents of Asian or European origin compared with a healthy population of the same origin (OR: 0.96; CI: 0.87–1.06; and *p* = 0.38 and OR: 1.03; CI: 0.93–1.14; and *p* = 0.34, respectively). However, children and adolescents with ALL originating from South American countries had the C allele sequences (AC and CC) more frequently compared with a healthy population of the same origin (OR: 1.48; CI: 1.15–1.89; *p* = 0.002) (Figure 5).

**Figure 6:**
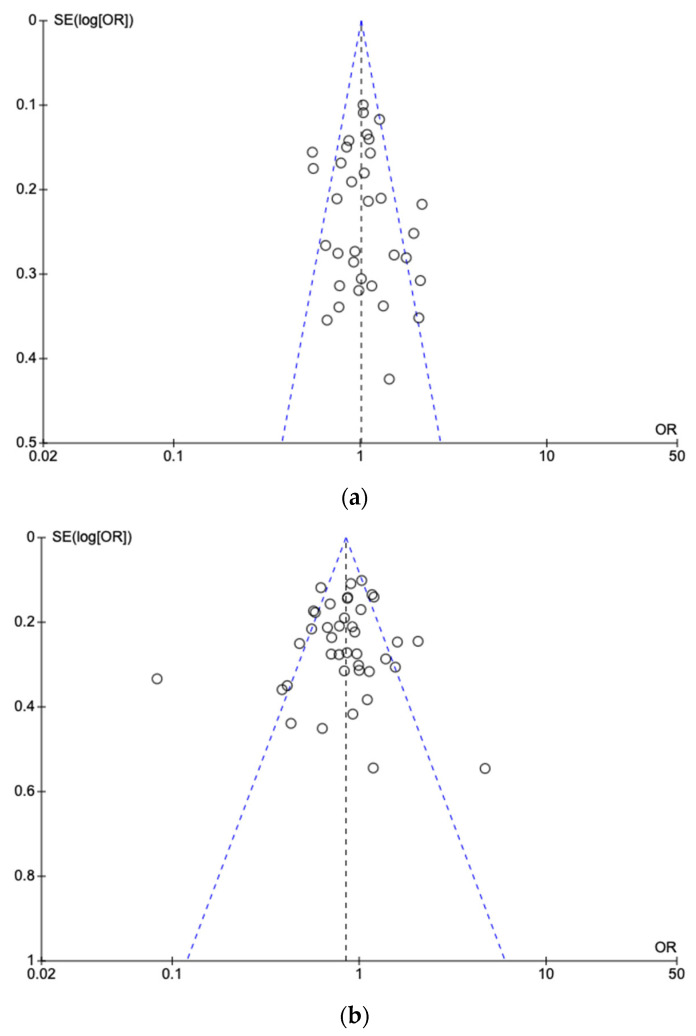
Funnel plot for the assessment of publication bias for the correlation of (**a**) T allele sequences (CT and TT variations) of the 677C>T polymorphism of the *MTHFR* gene in children and adolescents with ALL versus a healthy population and (**b**) C allele sequences (AC and CC variations) of the 1298A>C polymorphism of the *MTHFR* gene in children and adolescents with ALL versus a healthy population. Individual studies are represented by circles, the blue diagonal dashed lines represent the confidence interval 95%, while the black vertical dashed line represents the overall effect.

The analysis of publication bias for both polymorphisms (677C>T and 1298A>C) showed no significant bias, with symmetric diagrams in the funnel plot analysis, which was further verified using Egger’s regression test (*p* > 0.05) (Figure 6).

### 3.2. Acute Myeloblastic Leukemia (AML)

Four studies involving children and adolescents with AML were eligible and included in the present review, synthesizing data from a total of 339 children and adolescents in the patient groups and 1414 participants in the control groups. Children and adolescents with AML, when compared with a healthy population, did not present a difference in the studied prevalence of *MTHFR* polymorphisms (677C>T and 1298A>C). The only exception was the case–control study by Lightfoot et al. [86], in which 89 children and adolescents were included in the patient group, concluding that children and adolescents with AML had the CT variant of the 677C>T polymorphism less frequently compared with the control group (Table 1).

### 3.3. Central Nervous System (CNS) Tumors

We identified eight studies that included children and adolescents with eligible central nervous system (CNS) tumors. Three studies on various types of CNS tumors [107,108,109], which included a total of 678 children and adolescents in the patient groups, showed that there was no statistically significant difference in the frequency of *MTHFR* polymorphisms (677C>T and 1298A>C) compared with that in a healthy population. The study by De Miranda et al. [110], which included 29 children and adolescents with neuroblastomas in the patient group, did not show a statistically significant difference in the frequency of the 677C>T polymorphism compared with that in 92 healthy participants. Finally, we included four other eligible studies using our protocol that included 326 children and adolescents with retinoblastomas and 490 participants in the control groups [111,112,113,114]. The study by Soleimani et al. [112] reported that 96 children and adolescents with retinoblastomas had the 677C>T polymorphism less frequently, as both heterozygous and homozygous variants were recorded less frequently (OR: 0.51 and *p* = 0.031 and OR: 0.23 and *p* = 0.025, respectively). On the contrary, the study by Bisht et al. [113] reported that the SNPs 677C>T and also 1298A>C were associated with an increased incidence of retinoblastoma in children and adolescents, as CT variants were reported at a lower frequency in the control group (ORs: 16.02 and 10.2, respectively; *p* < 0.0001). The other two eligible studies [111,114] did not show a statistically significant association between *MTHFR* polymorphisms (677C>T and 1298A>C) and the incidence of retinoblastoma (Table 2).

### 3.4. Other Types of Cancer

Three eligible studies were identified that included children and adolescents with other types of cancer. The study by Stanulla et al. [115], which included 487 children and adolescents in the patient group with non-Hodgkin lymphoma, showed that the 677C>T polymorphism of the *MTHFR* gene did not appear at a different frequency compared to that in the healthy population. The study by Patîno-Garcia et al. [116], which included 96 children and adolescents with osteosarcomas, showed that the *MTHFR* polymorphisms (677C>T and 1298A>C) did not have a statistically significant difference in frequency compared to that in the healthy population. Finally, the study by Ferrara et al. [117], which included 34 children and adolescents with Wilms’ tumors, showed that the TT variant of the 677C>T polymorphisms occurs more frequently compared to its frequency in the healthy population (Table 3).

### 3.5. Quality Assessment of the Included Studies

This study conducted a quality assessment of 53 original case–control studies included in the systematic review and the meta-analysis. The studies included were assessed as high-quality, as they scored 7 or higher on the star-based system according to the Newcastle–Ottawa scale (NOS) (Table S3 in Supplementary Materials).

## 4. Discussion

The primary outcome of the present systematic review and meta-analysis was to present the incidence of *MTHFR* polymorphisms among children and adolescents with any type of cancer. The vast majority of eligible studies included children and adolescents with ALL. A total of 40 studies in our analysis included data from 7704 children and adolescents in the patient groups and 10,825 in the control groups. Children and adolescents with ALL appeared to have T allele sequences (CT and TT variants) of the 677C>T polymorphism of the *MTHFR* gene less frequently when compared with the healthy population. The suggested protective effect of the 677C>T polymorphism in children and adolescents with ALL contradicts the known association between this polymorphism, hyperhomocysteinemia and cancer. However, this protective effect has also been reported in the association of *MTHFR* polymorphisms with gliomas and *MTHFR* polymorphisms with hypothyroidism [21,30]. No difference in the 1298A>C polymorphism of the *MTHFR* gene was investigated in children and adolescents with ALL compared with that in the healthy controls. The subgroup analysis, based on country and continent of origin, revealed that the T allele sequences (CT and TT variants) of the 677C>T polymorphism of the *MTHFR* gene were recorded less frequently in children and adolescents from the South American, Asian and European subgroups compared to healthy populations of the same origin.

The subgroup analysis of the 1298A>C polymorphism of the *MTHFR* gene showed that children and adolescents from South America had the C allele sequences (AC and CC variants) more frequently compared with a healthy population of the same origin. The results of the different studies occasionally contradicted each other, for which there are various possible explanations. This includes the influence of the type of population studied because of the difference between the results from the Asian and European studies. Based on numerous studies, it is acceptable to hypothesize that polymorphisms in the *MTHFR* gene, 677C>T and 1298A>C, are associated with variations in the susceptibility rates for childhood ALL in non-Asian populations. Polymorphisms in other folate-related genes (*MTRR*, *MTR* [*MS*], *TYMS* [*TS*], *SLC19A1* [*RFC1*], *NNMT* and *SHMT1*) are less clearly associated with susceptibility to ALL [118]. Generally, it is clear that susceptibility to childhood ALL is partly related to constitutional differences in folate gene polymorphisms. Among the four studies included on children and adolescents with AML, in the present systematic review, there was no difference in the frequency of either *MTHFR* gene polymorphisms (677C>T and 1298A>C) between the patients and controls, except for one case–control study [86], where the CT variant of the 677C>T polymorphism was less frequently reported in the patient group when compared with the control group. The eight studies included involving children and adolescents with CNS tumors in the present systematic review revealed contradictory findings. Among children and adolescents with retinoblastoma, both variants of the 677C>T polymorphism (CT and TT) were reported less frequently compared to their frequency in a healthy population [112], whereas the CT variant of 677C>T and the AC variant of the 1298A>C polymorphism appeared 10 times more frequently (even 16 times more for the CT variation of 677C>T) compared with their frequency in a healthy population in another protocol [113]. Finally, children and adolescents with Wilms’ tumors presented with the TT variant of the 677C>T polymorphisms more frequently compared to its frequency in a healthy population [117].

The present study represents a systematic review of all of the available literature, focusing on the association of *MTHFR* gene polymorphisms and cancer in children and adolescents in a comprehensive manner. Previously published systematic reviews and meta-analyses including children and adolescents with ALL have shown significant inconsistency in investigating the possible associations between ALL and *MTHFR* gene polymorphisms [42,43,118,119,120,121,122,123]. Many investigators have suggested that there is no association between the 677C>T polymorphism of the *MTHFR* gene and children and adolescents with ALL [42,44,120,121]. Others have suggested that there is a slightly increased incidence of the 677C>T polymorphism in children and adolescents with ALL, while others have suggested that its incidence is reduced [29,43,118,119,123]. Similar inconsistency has been observed in the findings of studies on the 1298A>C polymorphism [42,43,118,119,120,121,122]. The discrepancies between our study and similar studies on the same questions probably arise due to their inclusion of case–control studies with a mixed-age population, in contrast to the present study, in which the age of the population was specific. Our analysis is consistent with the meta-analysis performed 12 years earlier by Yan et al. [119] in 2012, which also included studies exclusively on children and adolescents and obtained similar results to the findings of this study. However, our findings include the incorporation of new data from more original case studies. Additionally, the hereby conducted subgroup analysis provides further insights into the association of *MTHFR* gene polymorphisms based on the population’s origin, with interesting findings regarding the geographical variation in the investigated differences in polymorphisms in children and adolescents with ALL.

A recent systematic review and meta-analysis by Gohari et al. examined the correlation between *MTHFR* gene polymorphisms (677C>T and 1298A>C) in children and adolescents and retinoblastoma when compared with healthy controls [124]. Its findings supported that the 677C>T polymorphism is associated with susceptibility to retinoblastoma. However, the current study did not perform a statistical analysis due to the small number of the studies that met the eligibility criteria and due to their high heterogeneity.

A systematic review and meta-analysis by Qin et al. investigated the association between the *MTHFR* gene polymorphisms (677C>T and 1298A>C) and patients with AML compared to their frequency in a healthy population [125]. The authors concluded that there was no association between the two groups; however, they included several studies that consisted of adults or mixed-age populations, and thus, their conclusions apply to all age groups and may differ from those for pediatric AML. A different systematic review and meta-analysis by Wang et al. examined the association of the *MTHFR* gene polymorphisms (677C>T and 1298A>C) and patients with non-Hodgkin lymphoma compared to a healthy population [126]. They also similarly included many adult studies with mixed populations, and therefore, their conclusions may not apply to the pediatric population.

Based on the synthesis performed of the previously mentioned evidence, it is well established that a correlation between *MTHFR* gene polymorphisms (677C>T and 1298A>C) and ALL among children and adolescents emerges, which is of high interest. For other types of cancer, more studies that include solely children and adolescents are needed in order to extract firm and valid conclusions.

*MTHFR* gene polymorphisms have attracted even greater attention in the medical community in recent years due to their association with methotrexate. Methotrexate is a widely used drug in a variety of cancer treatments. It inhibits the action of dihydrofolate reductase within the folate cycle, thereby reducing tetrahydrofolate levels [127]. *MTHFR* gene polymorphisms result in reduced levels of the active form of folic acid (5-MethylTHF). Research has examined the potential implication of *MTHFR* gene polymorphisms in the adverse events and toxicity of methotrexate as part of cancer treatment, providing a new perspective on the clinical implications of the *MTHFR* gene. Specifically, it has been reported that children and adolescents with ALL and a confirmed 677C>T polymorphism of the *MTHFR* gene are more prone to mucositis and gastrointestinal-related adverse events, liver damage and lower platelet counts after receiving high doses of methotrexate as part of cancer treatment protocols (in ALL and non-Hodgkin lymphoma) [128,129,130,131,132,133,134,135]. These findings reveal the *MTHFR* gene as a promising clinical tool when implementing cancer treatment protocols. The identification of *MTHFR* gene polymorphisms in children and adolescents provides a prognosis and early prediction of the aforementioned adverse events and is therefore related to overall morbidity.

The present analysis included studies that consisted exclusively of children and adolescents and did not include studies with mixed elderly populations, therefore strengthening the resulting findings. Additionally, this review presented the association of *MTHFR* gene polymorphisms by cancer type, providing a comprehensive overview across distinct oncological entities with different clinical and molecular backgrounds. The statistical analysis in the current study only compared wild-type sequences with mutant sequences in studies that included children and adolescents with ALL (CC vs. CT and TT for the 677C>T polymorphism and AA vs. AC and CC for the 1298A>C polymorphism). We only included studies published in the English language. Statistical analyses were not performed for any other cancer types due to the limited studies per category and due to the heterogeneity of the studies included.

## 5. Conclusions

The present analysis suggests an association between the 677C>T polymorphism of the *MTHFR* gene and a diagnosis of ALL among children and adolescents. Pediatric and adolescent ALL patients appear to have T allele sequences (CT and TT variants) less frequently compared to the healthy population. These findings were confirmed in our subgroup analysis of populations from Europe, Asia and South America. The 1298A>C polymorphism of the *MTHFR* gene does not show any difference between children and adolescents with ALL and healthy controls. However, in the subgroup analysis, children and adolescents with ALL that originated from South American countries have C allele sequences (AC and CC variations) of the 1298A>C polymorphism more frequently compared to the same healthy population. Children and adolescents with AML or CNS tumors do not show significant differences in the distribution of *MTHFR* polymorphisms compared with that in healthy controls. For children and adolescents with retinoblastoma, inconsistent and contradictory findings are encountered regarding the role of *MTHFR* gene polymorphisms. There are insufficient data to extract valid conclusions regarding the relationship between *MTHFR* gene polymorphisms and other types of cancer in childhood and adolescence.

## Figures and Tables

**Table 1:** *MTHFR* gene polymorphisms in children and adolescents with AML versus a healthy population.

Author	Year	Country of Origin	Patient Group (*n)*	Control Group (*n*)	677C>T Polymorphism			1298A>C Polymorphism		
					CC	CT	TT	AA	AC	CC
Ramos [105]	2006	Brazil	182	315	1	0.88 (0.58–1.32)	1.19 (0.62–2.27)	1	1.09 (0.72–1.65)	1.39 (0.66–2.93)
Bolufer [106]	2007	Spain	35	51	1	0.98 (0.64–1.50)	0.87 (0.51–1.51)	-	-	-
Lightfoot [86]	2010	U.K.	58	378	1	0.51 (0.3–0.87)	1 (0.50–2.01)	1	0.67 (0.40–1.12)	1.22 (0.60–2.48)
Silva [96]	2013	Brazil	33	224	1	1.8 (0.83–3.90)	2.1 (0.45–9.71)	1	0.55 (0.24–1.26)	0.33 (0.10–1.04)

Data are presented as odds ratio (confidence interval) per polymorphism.

**Table 2:** *MTHFR* gene polymorphisms in children and adolescents with CNS tumors versus a healthy population.

Author	Year	Country of Origin	Type of Cancer	Patient Group (*n*)	Control Group (*n*)	677C>T Polymorphism			1298A>C Polymorphism		
						CC	CT	TT	AA	AC	CC
Sirachainan [107]	2008	Thailand	Glial tumors +	73	205	1	1.2 (0.6–2.1)	2 (0.3–12.2)	1	1 (0.5–1.7)	1.6 (0.6–4.3)
Salnikova [108]	2013	Russia	Glial and embryonic tumors ++	284	456	1	0.86 (0.64–1.16)	0.80 (0.46–1.39)	-	-	-
Greenop [109]	2015	Australia	Non-specified brain tumors	321	552	1	0.95 (0.7–1.29)	0.83 (0.51–1.36)	1	0.95 (0.7–1.28)	1.24 (0.73–2.09)
De Miranda [110]	2014	Brazil	Neuroblastoma	29	92	1	1.45 (0.58–3.63)	1.76 (0.37–8.18)	-	-	-
Santos de Lima [111]	2010	Brazil	Retinoblastoma	72	97	1	1.76 (0.61–5.04)	1.82 (0.65–5.07)	1	0.99 (0.28–3.46)	1.46 (0.39–5.39)
Soleimani [112]	2016	Iran	Retinoblastoma	96	204	1	0.51 (0.3–0.87)	0.23 (0.03–0.91)	1	1.11 (0.65–1.91)	0.72 (0.34–1.49)
Bisht [113]	2018	India	Retinoblastoma	90	90	1	16.03 (8.9–28.8)	0	1	10.2 (5.6–18.58)	0
Gohari [114]	2019	Slovenia	Retinoblastoma	66	99	1	0.91 (0.48–1.70)	1.16 (0.46–2.94)	1	0.813 (0.41–1.5)	1.33 (0.42–4.17)

Data are presented as odds ratio (confidence interval) per polymorphism. + Astrocytoma, oligodendroglioma, ependymoma. ++ Astrocytoma, oligodendroglioma, brain stem glioma, ependymoma and medulloblastoma, primitive neuroectodermal brain tumors (PNETs), atypical teratoid rhabdoid tumors and pineoblastoma.

**Table 3:** *MTHFR* gene polymorphisms in children and adolescents with other types of cancer per category versus a healthy population.

Author	Year	Country of Origin	Type of Cancer	Patient Group (*n*)	Control Group (*n*)	677C>T Polymorphism			1298A>C Polymorphism		
						CC	CT	TT	AA	AC	CC
Stanulla [115]	2005	Germany	Non-Hodgkin lymphoma	487	379	1	1.26 (0.95–1.68)	1.32 (0.86–2.04)	1	-	-
Patîno–Garcia [116]	2008	Spain	Osteosarcoma	96	110	1	0.87 (0.47–1.6)	0.67 (0.3–1.5)	1	1.26 (0.7–2.3)	1.1 (0.36–3.1)
Ferrara [117]	2009	Italy	Wilms’ tumors	34	70	1	1.42 (0.62–3.27)	3.22 (1.13–9.15)	1	-	-

Data are presented as odds ratio (confidence interval) per polymorphism.

## Supplementary Materials

The following supporting information can be downloaded at https://www.mdpi.com/article/10.3390/children12010108/s1. Table S1: PRISMA 2020 Checklist of the systematic review; Table S2: Baseline Characteristics of studies included in the systematic review; Table S3: Newcastle-Ottawa scale of studies included in the systematic review.
