# Supplementary material for: MTHFR Gene Polymorphisms and Cancer Risk in Children and Adolescents: A Systematic Review and Meta-Analysis
Source: Children (Basel). 2025 Jan 17;12(1):108. doi: 10.3390/children12010108 (PMC11764102; doi:10.3390/children12010108)
Supplement: Supplementary file 1 [file children-12-00108-s001.zip › children-3396015-supplementary.pdf]

Supplementary Material

PRISMA 2020 Checklist

Table S1: PRISMA 2020 Checklist of the systematic review

Section and Topic	Item #	Checklist item	Location where item is reported
**TITLE**			Lines in text
Title	1	Identify the report as a systematic review.	2
**ABSTRACT**			
Abstract	2	See the PRISMA 2020 for Abstracts checklist.	13
**INTRODUCTION**			
Rationale	3	Describe the rationale for the review in the context of existing knowledge.	75-89
Objectives	4	Provide an explicit statement of the objective(s) or question(s) the review addresses.	90-92
**METHODS**			
Eligibility criteria	5	Specify the inclusion and exclusion criteria for the review and how studies were grouped for the syntheses.	94-98
Information sources	6	Specify all databases, registers, websites, organisations, reference lists and other sources searched or consulted to identify studies. Specify the date when each source was last searched or consulted.	102-104
Search strategy	7	Present the full search strategies for all databases, registers and websites, including any filters and limits used.	102-113
Selection process	8	Specify the methods used to decide whether a study met the inclusion criteria of the review, including how many reviewers screened each record and each report retrieved, whether they worked independently, and if applicable, details of automation tools used in the process.	116-122
Data collection process	9	Specify the methods used to collect data from reports, including how many reviewers collected data from each report, whether they worked independently, any processes for obtaining or confirming data from study investigators, and if applicable, details of automation tools used in the process.	114-124
Data items	10a	List and define all outcomes for which data were sought. Specify whether all results that were compatible with each outcome domain in each study were sought (e.g. for all measures, time points, analyses), and if not, the methods used to decide which results to collect.	120-125
	10b	List and define all other variables for which data were sought (e.g. participant and intervention characteristics, funding sources). Describe any assumptions made about any missing or unclear information.	120-124
Study risk of bias assessment	11	Specify the methods used to assess risk of bias in the included studies, including details of the tool(s) used, how many reviewers assessed each study and whether they worked independently, and if applicable, details of automation tools used in the process.	125-128
Effect measures	12	Specify for each outcome the effect measure(s) (e.g. risk ratio, mean difference) used in the synthesis or presentation of results.	150-153
Synthesis methods	13a	Describe the processes used to decide which studies were eligible for each synthesis (e.g. tabulating the study intervention characteristics and comparing against the planned groups for each synthesis (item #5)).	116-121
	13b	Describe any methods required to prepare the data for presentation or synthesis, such as handling of missing summary statistics, or data	127-130

Section and Topic	Item #	Checklist item	Location where item is reported
		conversions.	
	13c	Describe any methods used to tabulate or visually display results of individual studies and syntheses.	125-128, 144-145
	13d	Describe any methods used to synthesize results and provide a rationale for the choice(s). If meta-analysis was performed, describe the model(s), method(s) to identify the presence and extent of statistical heterogeneity, and software package(s) used.	143-149,
	13e	Describe any methods used to explore possible causes of heterogeneity among study results (e.g. subgroup analysis, meta-regression).	155-157
	13f	Describe any sensitivity analyses conducted to assess robustness of the synthesized results.	-
Reporting bias assessment	14	Describe any methods used to assess risk of bias due to missing results in a synthesis (arising from reporting biases).	131-135, table in suppl. material
Certainty assessment	15	Describe any methods used to assess certainty (or confidence) in the body of evidence for an outcome.	131-135, table in suppl. material
**RESULTS**			
Study selection	16a	Describe the results of the search and selection process, from the number of records identified in the search to the number of studies included in the review, ideally using a flow diagram.	Figure 1
	16b	Cite studies that might appear to meet the inclusion criteria, but which were excluded, and explain why they were excluded.	165- 166
Study characteristics	17	Cite each included study and present its characteristics.	166-168, Figures 2-5, tables 1-3, table in suppl. material
Risk of bias in studies	18	Present assessments of risk of bias for each included study.	Table in suppl. material, Figure 6
Results of individual studies	19	For all outcomes, present, for each study: (a) summary statistics for each group (where appropriate) and (b) an effect estimate and its precision (e.g. confidence/credible interval), ideally using structured tables or plots.	Figures 2-5
Results of syntheses	20a	For each synthesis, briefly summarise the characteristics and risk of bias among contributing studies.	174-278 Table in suppl.

Section and Topic	Item #	Checklist item	Location where item is reported
			material, Figure 6
	20b	Present results of all statistical syntheses conducted. If meta-analysis was done, present for each the summary estimate and its precision (e.g. confidence/credible interval) and measures of statistical heterogeneity. If comparing groups, describe the direction of the effect.	Figures 2-5
	20c	Present results of all investigations of possible causes of heterogeneity among study results.	Figures 2-5
	20d	Present results of all sensitivity analyses conducted to assess the robustness of the synthesized results.	-
Reporting biases	21	Present assessments of risk of bias due to missing results (arising from reporting biases) for each synthesis assessed.	211-213, Figure 6
Certainty of evidence	22	Present assessments of certainty (or confidence) in the body of evidence for each outcome assessed.	274-278, Table in suppl. material
**DISCUSSION**			
Discussion	23a	Provide a general interpretation of the results in the context of other evidence.	280-320
	23b	Discuss any limitations of the evidence included in the review.	384-390
	23c	Discuss any limitations of the review processes used.	384-390
	23d	Discuss implications of the results for practice, policy, and future research.	339-342<br>359- 363,<br>375-379
**OTHER INFORMATION**			
Registration and protocol	24a	Provide registration information for the review, including register name and registration number, or state that the review was not registered.	99-100
	24b	Indicate where the review protocol can be accessed, or state that a protocol was not prepared.	99-100
	24c	Describe and explain any amendments to information provided at registration or in the protocol.	-
Support	25	Describe sources of financial or non-financial support for the review, and the role of the funders or sponsors in the review.	412
Competing interests	26	Declare any competing interests of review authors.	416
Availability of data, code and other materials	27	Report which of the following are publicly available and where they can be found: template data collection forms; data extracted from included studies; data used for all analyses; analytic code; any other materials used in the review.	-

From: Page MJ, McKenzie JE, Bossuyt PM, Boutron I, Hoffmann TC, Mulrow CD, et al. The PRISMA 2020 statement: an updated guideline for reporting systematic reviews. BMJ 2021;372:n71. doi: 10.1136/bmj.n71. This work is licensed under CC BY 4.0. To view a copy of this license, visit https://creativecommons.org/licenses/by/4.0/

Table S2: Baseline Characteristics of studies included in the systematic review

Author, year	Population's origin	Patients' sample size	Controls' sample size	Gender (Female/ Male) patients' groups	Gender (Female/ Male) controls' group	Age in years (mean value or range) patients' group	Age in years (mean value or range) controls' group	Method of genetic analysis
Alcasabas [65], 2008	Philippines	295	394	118/177	185/209	6.9	-	qPCR - TaqMan
Azhar [66], 2012	Kurdish	72	109	24/48	46/63	8.1	8.1	PCR - RFLP
Bahari [67], 2016	Iran	100	120	42/58	64/56	6.2	5.8	PCR - RFLP
Balta [68], 2003	Turkey	142	185	48/96	65/120	6.8	7.4	PCR - RFLP
Bohanec [69], 2007	Slovenia	68	258	41/27	113/145	4.6	24	PCR - RFLP
Chan [70], 2011	Indonesia	185	177	78/107	73/104	5.2	-	PCR - RFLP
Chatzidakis [71], 2006	Greece	52	88	29/23	36/52	5.3	19 -77	PCR - RFLP
Damnjanovic [72], 2010	Serbia	78	412	-	-	1 - 14	-	PCR - RFLP
De Jonge [73], 2009	Netherlands	245	496	98/147	-	-	--	PCR - RFLP
Feng [74], 2012	China	45	45	-	-	1 - 18	1 -18	PCR - RFLP
Franco [75], 2001	Brazil	71	71	43/28	43/28	7.6	7.6	PCR - RFLP
Giovannetti [76], 2008	Indonesia	70	44	34/36	18/26	6.1	--	qPCR - TaqMan
Gómez-Gómez [77], 2019	Mexico	60	60	27/33	34/26	5.9	9.9	PCR - RFLP
Gutiérrez-Álvarez [78], 2016	Mexico	70	152	29/41	64/88	6.7	6.7	qPCR
Heuvel-Eibrink [79], 2011	Rotterdam	68	148	21/47	28/71	6.4	8.1	PCR - RFLP
Kałużna [80], 2017	Poland	117	404	48/69	208/196	10.4	37.7	qPCR - TaqMan
Karathanasis [81], 2011	Greece	35	48	14/21	-	6.3	-	PCR - RFLP
Kim [82], 2006	South Korea	66	100	27/39	-	9.0	-	ASO hybridization
Krajinovic [83], 2004	Canada	270	300	113/157	-	4.9	-	ASO hybridization
Kreile [84], 2014	Poland	68	102	33/35	-	4.9	-	PCR - RFLP
Li [85], 2014	China	98	93	44/54	-	5.1	5.7	PCR - RFLP
Lightfoot [86], 2010	U.K.	939	824	421/518	378/446	0 - 14	-	PCR - RFLP
Metayer [87], 2011	Taiwan	377	448	177/200	211/237	6.1	-	PCR - RFLP

Milne [88], 2015	Australia	392	535	216/176	283/252	0 - 15	0 - 15	qPCR - TaqMan
Mosaad [89], 2015	Egypt	100	100	39/61	41/59	7.8	7.5	PCR - RFLP
Nikbaht [90], 2012	India	125	100	28/97	23/77	6.4	6.5	PCR - RFLP
Oliveira [91], 2005	Portugal	103	111	-	-	1 - 16	25	PCR - RFLP
Pei [92], 2015	Taiwan	266	266	118/148	118/148	8.4	5.2	PCR - RFLP
Reddy [93], 2006	India	135	142	48/87	-	4.2	4.2	PCR - RFLP
Sadananda [94], 2010	India	86	99	27/59	28/71	7.4	8.1	PCR - RFLP
Schnakenberg [95], 2005	Germany	433	379	175/268	-	1 - 18	18 - 68	PCR - RFLP
Silva [96], 2013	Brazil	144	224	-	-	1 - 19	1 - 19	PCR - RFLP
Sood [97], 2010	India	95	255	25/70	-	5.5	25	PCR - RFLP
Thirumaran [98], 2005	Germany	460	1472	-	-	6.9	-	qPCR - TaqMan
Tong [99], 2010	China	361	508	145/216	190/318	1-18	-	PCR - RFLP
Wiemels [100], 2001	U.K.	253	200	-	-	1-15	1 - 18	ASO hybridization
Xia [101], 2017	China	210	423	118/92	225/198	8.4	8.1	PCR - RFLP
Yeoh [102], 2010	China	318	345	136/185	190/156	5.6	-	PCR - RFLP
Zanrosso [103], 2006	Brazil	176	199	84/92	116/83	6.2	25	PCR - RFLP
Zou [104], 2017	China	79	102	43/36	39/63	1 - 15	13.5	qPCR
Ramos [105], 2006	Brazil	182	315	93/89	164/151	7.1	5.4	PCR - RFLP
Bolufer [106], 2007	Spain	35	51	-	-	0 - 16	0 - 16	qPCR
Sirachainan [107], 2008	Thailand	73	205	31/42	98/107	7.7	4.2	PCR - RFLP
Salnikova [108], 2013	Russia	284	464	124/160	187/277	7.7	27.5	qPCR
Greenop [109], 2015	Australia	321	552	132/189	259/293	0 - 15	0 - 15	qPCR - TaqMan
De Miranda [110], 2014	Brazil	29	92	14/15	52/40	2.6	11	PCR - RFLP
Santos de Lima [111], 2010	Brazil	72	97	33/39	52/46	2.8	10	PCR - RFLP
Soleimani [112], 2016	Iran	96	204	-	-	1 - 6	6 - 18	PCR - RFLP
Bisht [113], 2018	India	90	90	41/49	37/53	2.2	8.7	PCR - RFLP
Gohari [114], 2019	Iran	66	99	-	-	0 - 18	0 -18	qPCR

Stanulla [115], 2005	Germany, Austria	487	379	144/343	-	0 - 18	0 - 18	qPCR
Patîno-Garcia [116], 2008	Spain	96	110	55/41	-	14.5	--	qPCR - TaqMan
Ferrara [117], 2009	Italy	34	70	-	-	3.2 – 5.4	-	PCR - RFLP

Abbreviations: PCR- RFLP: Polymerase Chain Reaction- Restriction Fragment Length Polymorphism, qPCR: quantitative Polymerase Chain Reaction, ASO hybridization: Allele Specific Oligonucleotide Hybridization

Table S3: Newcastle-Ottawa scale of studies included in the systematic review

**Studies**	**Selection**				**Comparability**	**Exposure**			**Total Quality Score**
Author, year	Adequate definition of cases	Representativeness of the cases	Selection of controls	Definition of controls	Comparability of cases and controls	Ascertainment of exposure	Same method of ascertainment for cases and controls	Non-Respone Rate	
Alcasabas [65] 2008	✸	✸	-	✸	✸✸	✸	✸	✸	8
Azhar [66], 2012	✸	✸	✸	✸	✸✸	✸	✸	✸	9
Bahari [67], 2016	✸	✸	✸	✸	✸✸	✸	✸	-	8
Balta [68], 2003	✸	✸	✸	✸	✸✸	✸	✸	-	9
Bohanec [69], 2007	✸	✸	✸	-	✸✸	✸	✸	✸	8
Chan [70], 2011	✸	✸	✸	✸	✸✸	✸	✸	✸	9
Chatzidakis [71] 2006	✸	✸	✸	-	✸✸	✸	✸	✸	8
Damnjanovic [72], 2010	✸	✸	✸	✸	✸✸	✸	✸	✸	9
De Jonge [73] 2009	✸	✸	✸	-	✸✸	✸	✸	✸	8
Feng [74], 2012	✸	✸	✸	✸	✸✸	✸	✸	✸	9
Franco [75], 2001	✸	✸	✸	-	✸✸	✸	✸	✸	8
Giovannetti [76], 2008	✸	✸	✸	-	✸✸	✸	✸	✸	8
Gómez-Gómez [77], 2019	✸	✸	✸	✸	✸✸	✸	✸	✸	9
Gutiérrez-Álvarez [78], 2016	✸	✸	✸	-	✸✸	✸	✸	✸	8
Heuvel- Eibrink [79], 2011	✸	✸	✸	✸	✸✸	✸	✸	✸	9
Kaluzna [80], 2017	✸	✸	✸	✸	✸✸	✸	✸	✸	9

Karathanasis [81], 2011	✸	✸	✸	✸	✸✸	✸	✸	✸	9
Kim [82], 2006	✸	✸	✸	✸	✸✸	✸	✸	✸	9
Krajinovi [83] 2004	✸	✸	✸	✸	✸✸	✸	✸	✸	9
Kreile [84], 2014	✸	✸	✸	✸	✸✸	✸	✸	✸	9
Li [85], 2014	✸	✸	✸	-	✸✸	✸	✸	✸	8
Lightfoot [86] 2010	✸	✸	✸	-	✸✸	✸	✸	✸	8
Metayer [87], 2011	✸	✸	✸	✸	✸✸	✸	✸	-	8
Milne [88], 2015	✸	✸	✸	-	✸✸	✸	✸	✸	8
Mosaad [89], 2015	✸	✸	✸	✸	✸✸	✸	✸	✸	9
Nikbaht [90], 2012	✸	✸	✸	✸	✸✸	✸	✸	✸	9
Oliveira [91], 2005	✸	✸	✸	✸	✸✸	✸	✸	✸	9
Pei [92], 2015	✸	✸	✸	✸	✸✸	✸	✸	✸	9
Reddy [93], 2006	✸	✸	✸	-	✸✸	✸	✸	✸	8
Sadananda [94] 2010	✸	-	✸	-	✸✸	✸	✸	✸	7
Schnakenberg [95], 2005	✸	✸	✸	✸	✸✸	✸	✸	✸	9
Silva [96], 2013	✸	✸	✸	-	✸	✸	✸	✸	7
Sood [97], 2010	✸	✸	✸	✸	✸✸	✸	✸	✸	9
Thirumaran [98], 2005	✸	-	✸	-	✸✸	✸	✸	✸	7
Tong [99], 2010	✸	✸	✸	✸	✸✸	✸	✸	✸	9
Wiemels [100] 2001	✸	✸	✸	-	✸✸	✸	✸	✸	8
Xia [101], 2017	✸	✸	✸	✸	✸✸	✸	✸	✸	9
Yeoh [102], 2010	✸	✸	✸	-	✸	✸	✸	✸	7
Zanrosso[103] 2006	✸	✸	✸	✸	✸✸	✸	✸	✸	9
Zou [104], 2017	✸	✸	✸	-	✸✸	✸	✸	✸	8
Ramos [105], 2006	✸	✸	✸	✸	✸✸	✸	✸	✸	9
Bolufer [106], 2007	✸	✸	✸	✸	✸✸	✸	✸	✸	9
Sirachainan [107] 2008	✸	✸	✸	-	✸✸	✸	✸	✸	8
Salnikova [108] 2013	✸	✸	✸	✸	✸✸	✸	✸	✸	9
Greenop [109] 2015	✸	✸	✸	✸	✸✸	✸	✸	-	8
De Miranda [110], 2014	✸	-	✸	✸	✸✸	✸	✸	✸	8
Santos de Lima [111], 2010	✸	✸	✸	✸	✸✸	✸	✸	✸	9

Soleimani [112] 2016	✸	-	✸	✸	✸✸	✸	✸	✸	8
Bisht [113], 2018	✸	✸	✸	✸	✸✸	✸	✸	✸	9
Gohari [114], 2019	✸	✸	✸	✸	✸✸	✸	✸	-	8
Stanulla [115] 2005	✸	✸	✸	✸	✸	✸	✸	✸	8
Patîno-Garcia [116], 2008	✸	✸	✸	✸	✸✸	✸	✸	✸	9
Ferrara [117], 2009	✸	-	✸	✸	✸	✸	✸	✸	7

Each study was able to receive up to one star (✸) for each of the subcategories in the Selection and Exposure categories. Two stars could be given for the Comparability category.
